# Molecular analysis of the integrons of metallo-β-lactamase-producing *Pseudomonas aeruginosa* isolates collected by nationwide surveillance programs across Japan

**DOI:** 10.1186/s12866-015-0378-8

**Published:** 2015-02-21

**Authors:** Yoko Mano, Tomoo Saga, Yoshikazu Ishii, Ayumi Yoshizumi, Robert A Bonomo, Keizo Yamaguchi, Kazuhiro Tateda

**Affiliations:** Department of Microbiology and Infectious Diseases, Toho University School of Medicine, 5-21-16 Omori-nishi, Ota-ku, Tokyo, 143-8540 Japan; Research Service, Louis Stokes Cleveland Department of Veterans Affairs Medical Center, 10701 East Blvd., Cleveland, OH 44106 USA; Departments of Medicine, Pharmacology, Molecular Biology and Microbiology, Case Western Reserve University School of Medicine, Cleveland, OH 44106 USA

**Keywords:** Metallo-β-lactamases, Integron, *Pseudomonas aeruginosa*

## Abstract

**Background:**

We investigate the evolving molecular epidemiology of metallo-β-lactamase (MBL)-producing *Pseudomonas aeruginosa* isolates collected in a 100 institution, nationwide surveillance study in Japan from 2004 to 2006.

**Results:**

MBL-producers were detected in 23/996 isolates (2.3%) in 2004 and 21/992 (2.1%) in 2006. Antimicrobial resistance (specifically, carbapenem resistance) rates between two periods did not differ significantly. MBL-producers were more prevalent in urinary tract isolates. *bla*_IMP-1_ group was the most predominant (38 isolates, 80%), followed by 3 *bla*_IMP-7_, 2 *bla*_IMP-11_ group, and 1 *bla*_VIM-1_. All MBL genes were identified in 16 different class 1 integrons, most of which were novel to INTEGRALL database. A total of 17 isolates of sequence type (ST) 235, a recognized worldwide drug-resistant lineage, were distributed in 5 geographic regions across Japan. ST235 isolates included a sublineage associated with In113-like integron. ST357 was identified in 14 isolates, 9 of which harboring a sole *bla*_IMP-1_ gene cassette (In994) were recovered from Chugoku region in 2004. ST357 isolates with *bla*_IMP-11_ group or ST235 with *bla*_IMP-7_ emerged in 2006. We also report for the first time the presence of novel *fosI* gene cassette in strains other than *Mycobacterium* spp.

**Conclusions:**

Our data give an important “snapshot” of the molecular characteristics and dynamics of MBL-producing lineages in *P. aeruginosa* in Japan. The significant association of specific genotypes and integrons implies that dissemination and transmission of the preexisting resistant lineage, rather than horizontal gene transfer *in situ*, might largely explain their endemicity.

## Background

As carbapenems are among the most reliable therapeutic options for treating *Pseudomonas aeruginosa* infection, the finding of carbapenem resistance is an ominous development that challenges this “last resort antibiotic”. Production of metallo-β-lactamases (MBLs) is an important mechanism of carbapenem resistance, not only because MBLs can hydrolyse most β-lactams including carbapenems, but MBL-producing *P. aeruginosa* are frequently also multidrug-resistant (resistant to ≥ 3 classes) [1]. More importantly, *bla*_MBL_ genes are often located in the gene cassette (GC) arrays in integrons, where multiple resistant determinants against antimicrobials or disinfectants are accumulated, and can be horizontally transferred [2].

Multilocus sequence typing (MLST), a genotyping method based on nucleotide sequencing of several house-keeping genes in the bacterial chromosome, has been increasingly applied for epidemiological investigation of pathogenic microorganisms [3]. In *P. aeruginosa* isolates, international clones such as ST (sequence type) 235, ST244, ST175, and ST357 are reported to have widespread dissemination [4-11]. Most notably, ST235 and their close variants (e.g., clonal complex 235) appear prominent in multiple-drug resistance.

Recently, the INTEGRALL database was developed for providing a systematic nomenclature for integrons [12]; these genetic elements can be potentially shared by a wide range of microorganisms as a result of horizontal gene transfer [13]. Moreover, GC arrays of integrons could be excised or integrated by the action of integrases and therefore be more diverse [2]. Therefore, INTEGRALL is expected to facilitate our comprehensive understanding of the complexity and development of integron-mediated drug resistance.

In Japan, as many as 80-90% of the MBL-producing *P. aeruginosa* were reported to be resistant to carbapenems, aminoglycosides, and quinolones [14,15]. Notably, several molecular epidemiological surveys report the increasing prevalence of multidrug-resistant *P. aeruginosa* (MDRP); in these the predominance of IMP-type MBL genes as well as ST235 and ST357 among MDRP is documented [15-20]. Although the prevalence of MBL among *P. aeruginosa* clinical isolates was reported as 2-4% [21,22], molecular epidemiology of MBL-producing *P. aeruginosa* in consecutive nationwide surveillance programs has not been analysed.

In the current study, we conducted a molecular epidemiological survey of the genetic context of MBL-producing *P. aeruginosa* isolates collected by nationwide surveillance. Our data not only provide an important “snapshot” of the molecular characteristics and dynamics of MBL-producing predominant lineages including ST235 and ST357, but also provide important baseline information for future surveillance programs. (This work was presented in part at the 51st Interscience Conference on Antimicrobial Agents and Chemotherapy, Chicago, IL, 2011.)

## Results and discussion

### Antimicrobial susceptibility of MBL-producing *P. aeruginosa* isolates

In the present study, 44 MBL-producing *P. aeruginosa* isolates were identified (Table 1); these were recovered from 6 out of 8 geographic regions across Japan. The number of isolates is comparable in 2004 and in 2006. MBL-producers were isolated more frequently from urinary tract specimens than from other specimens (24/408 vs. 20/1580; *P* < 0.0001). Although this trend was consistent with the previous reports in Japan [23], the reason for preference of MBL-producers for urinary tract was not addressed in this study where we did not analyse non-MBL-producers.

**Table 1:** **Distribution of metallo-β-lactamase-producing** ***Pseudomonas aeruginosa*** **across clinical specimens in the present study** ^**a**^

	**Year of isolation**	
**Clinical specimens**	**’04**	**’06**
Urinary tract	9/188 (0.9%)	15/220 (1.5%)
Respiratory tract	2/491 (0.2%)	2/449 (0.2%)
Gastrointestinal tract	1/33 (0.1%)	2/62 (0.2%)
Blood and fluids	2/73 (0.2%)	0/41 (0%)
Others	9/211 (0.9%)	2/220 (0.2%)
Total	23/996 (2.3%)	21/992 (2.1%)

^a^shown as a number of metallo-β-lactamase-producing *P. aeruginosa* isolates divided by a number of all *P. aeruginosa* isolates.

All MBL-producers met the international criteria for MDRP (Table 2). Overall, the antibiotic resistance patterns of MBL-producers did not differ significantly among the isolates between 2004 and 2006, except in cases where piperacillin was tested (4/23 resistant to piperacillin in 2004 vs 16/21 in 2006; *P* = 0.0002). Although this result reinforces the impact of the MBL production in MDRP, their limited susceptibility to the entire armamentarium of β-lactams including piperacillin or aztreonam, which MBLs are expected to hydrolyse less efficiently, suggests the involvement of multiple factors contributing to their drug resistance [24].

**Table 2:** **Profiles of metallo-β-lactamase-producing** ***Pseudomonas aeruginosa*** **in this study**

**No.**	**Year**	**Location**	**Specimens**	**PFGE**	**ST**	**Integron**		**MIC (μg/ml)**							
**TUM**						**No**	**GC arrays**	**PIPC**	**CAZ**	**CFPM**	**AZT**	**IPM**	**AMK**	**ABK**	**CPFX**
4046	2004	Shikoku	Urine	A	1070	In113	*bla*_IMP-1_ | *aacA28* | *aadA1*	32	>512	>512	32	512	256	4	16
3986	2006	Kanto	Urine	C	235	(partial)^a^	*bla*_IMP-7_^a^	128	512	256	8	64	>512	32	16
3995	2006	Kanto	Urine	C	235	(partial)^a^	*bla*_IMP-7_^a^	256	>512	256	8	64	>512	64	16
3997	2006	Kanto	Purulent discharge	C	235	(partial)^a^	*bla*_IMP-7_^a^	8	>512	256	4	512	>512	64	32
3988	2006	Kinki	Urine	A	235	In113	*bla*_IMP-1_ | *aacA28* | *aadA1a*	256	>512	>512	64	64	256	2	32
3977	2006	Hokkaido	Feces	B	235	In113	*bla*_IMP-1_ | *aacA28* | *aadA1a*	256	512	512	64	128	256	2	32
3978	2006	Chugoku	Drain	B	235	In113	*bla*_IMP-1_ | *aacA28* | *aadA1a*	256	512	256	16	64	256	4	32
3984	2006	Kanto	Urine	B	235	In113	*bla*_IMP-1_ | *aacA28* | *aadA1a*	256	512	256	16	64	>512	2	>128
3989	2006	Chugoku	Urine	B	235	In113	*bla*_IMP-1_ | *aacA28* | *aadA1a*	256	>512	>512	64	64	>512	4	32
3990	2006	Kanto	Sputum	B	235	In113	*bla*_IMP-1_ | *aacA28* | *aadA1a*	128	>512	256	8	64	>512	4	>128
3991	2006	Kanto	Urine	B	235	In113	*bla*_IMP-1_ | *aacA28* | *aadA1a*	256	512	256	8	64	>512	4	>128
3994	2006	Kanto	Urine	B	235	In113	*bla*_IMP-1_ | *aacA28* | *aadA1a*	128	512	256	32	512	>512	2	32
4029	2004	Hokkaido	Feces	B	235	In113	*bla*_IMP-1_ | *aacA28* | *aadA1a*	32	256	256	16	256	128	1	16
4034	2004	Kanto	Purulent discharge	B	235	In113	*bla*_IMP-1_ | *aacA28* | *aadA1a*	128	>512	256	16	128	>512	4	16
4035	2004	Chugoku	Urine	B	235	In113	*bla*_IMP-1_ | *aacA28* | *aadA1a*	512	>512	512	32	64	256	2	16
4031	2004	Kanto	Purulent discharge	B	235	In915	*bla*_IMP-6_ | *aacA28* | *aadA1a*	64	128	128	16	>512	>512	4	32
3993	2006	Shikoku	Urine	B	235	In829	*bla*_IMP-10_ | *aacA28* | *aadA1a*	16	>512	512	>512	256	128	2	>128
4036	2004	Kanto	Urine	B	235	In829	*bla*_IMP-10_ | *aacA28* | *aadA1a*	256	>512	256	16	512	>512	4	32
3992	2006	Chugoku	Urine	D	357	In994	*bla*_IMP-1_	32	>512	>512	64	128	64	1	>128
4033	2004	Chugoku	Urine	D	357	In994	*bla*_IMP-1_	64	>512	>512	32	512	8	0.5	>128
4038	2004	Chugoku	Purulent discharge	D	357	In994	*bla*_IMP-1_	64	>512	>512	256	256	8	≤0.25	32
4039	2004	Chugoku	Urine	D	357	In994	*bla*_IMP-1_	32	>512	>512	256	256	8	≤0.25	64
4040	2004	Chugoku	Urine	D	357	In994	*bla*_IMP-1_	16	512	512	64	256	64	1	>128
4041	2004	Chugoku	Purulent discharge	D	357	In994	*bla*_IMP-1_	16	512	512	64	>512	256	1	>128
4042	2004	Chugoku	Urine	D	357	In994	*bla*_IMP-1_	16	512	512	32	256	64	1	>128
4043	2004	Chugoku	Feces	D	357	In994	*bla*_IMP-1_	16	512	512	32	>512	256	0.5	>128
4044	2004	Chugoku	Sputum	D	357	In994	*bla*_IMP-1_	32	512	512	64	>512	256	1	>128
4045	2004	Chugoku	Urine	D	357	In994	*bla*_IMP-1_	32	512	512	64	128	64	1	>128
3996	2006	Chugoku	Pharyngeal mucus	D	357	In995	*bla*_IMP-10_	8	>512	>512	32	256	64	1	>128
3979	2006	Shikoku	Urine	D	357	In826	*aacA7* | *fosI* | *bla*_IMP-41_ | *qacG*	8	>512	512	8	256	256	8	>128
3985	2006	Kanto	Feces	D	357	In991	*aacA7* | *bla*_IMP-11_ | *bla*_OXA-2_ | *qacG*	256	>512	256	8	64	128	2	16
4049	2004	Kyusyu	Urine	E	357	In833	*bla*_IMP-6_ | *fosE*	2	64	64	4	32	8	≤0.25	8
4047	2004	Kyusyu	Purulent discharge	F	244	In994	*bla*_IMP-1_	64	512	128	8	4	>512	32	>128
4048	2004	Kyusyu	Purulent discharge	F	244	In994	*bla*_IMP-1_	64	512	128	8	4	>512	32	>128
4037	2004	Kinki	Urine	F	244	In832	*fosE* | *aacA31* | *bla*_VIM-2_	64	64	16	16	>512	256	1	16
3980	2006	Kyusyu	Urine	G	244	In993	*bla*_IMP-10_ | *aadA2* | *bla*_OXA-47b_	128	>512	512	16	8	16	≤0.25	32
4028	2004	Hokkaido	Urine	H	277	In994	*bla*_IMP-1_	512	>512	>512	64	256	16	0.5	>128
4030	2004	Hokkaido	Urine	H	277	In831	*bla*_IMP-10_ | *aacA1*, *gcuG*	16	>512	>512	64	>512	>512	8	32
4027	2004	Hokkaido	Sputum	I	186	In830	*bla*_IMP-1_ | *aacA4′-3* | *aacA1*, *gcuG* | *aadA1a*	32	512	128	16	4	128	8	8
3981	2006	Kyusyu	Urine	J	175	In827	*bla*_IMP-1_ | *aacA4′-3* | *aacA4′-3* | *catB6* | *bla*_CARB-12_	512	>512	>512	128	256	128	4	>128
3982	2006	Kyusyu	Urine	J	175	In827	*bla*_IMP-1_ | *aacA4′-3* | *aacA4′-3* | *catB6* | *bla*_CARB-12_	>512	>512	>512	128	256	64	4	32
3983	2006	Kyusyu	Urine	J	175	In827	*bla*_IMP-1_ | *aacA4′-3* | *aacA4′-3* | *catB6* | *bla*_CARB-12_	512	>512	>512	128	256	64	8	32
4032	2004	Kanto	Blood	J	308	In992	*aacA7* | *bla*_IMP-1_	16	>512	512	16	256	32	0.5	8
3987	2006	Kyusyu	Urine	K	360	In828	*bla*_IMP-1_ | *aadA24*, *aacA4′-3*	128	256	128	32	64	128	2	32

PFGE, pulsed-field gel electrophoresis; ST, sequence type; PIPC, piperacillin; CAZ, ceftazidime; CFPM, cefepime; AZT, aztreonam; IPM, imipenem; AMK, amikacin; ABK, arbekacin; CPFX, ciprofloxacin.

^a^3′-conserved segment (3’CS) was unknown and therefore no integron number was attributed.

Acknowledging that CLSI breakpoints were not available, the majority of MBL-producers showed increased susceptibility to arbekacin (MIC >8 μg/ml in only 5 strains, 11%). Although further investigation is needed, this agent might be a candidate for consideration as a therapeutic agent [25]. For more than two decades in Japan, arbekacin is a commercially available aminoglycoside effective against methicillin-resistant *Staphylococcus aureus* infections and its use in Gram negative infections is being explored .

### MBL genes and class 1 integrons among MBL-producing *P. aeruginosa*

Among MBL-producing *P. aeruginosa* isolates, seven different MBL genes were identified. The most prevalent was *bla*_IMP-1_ group found in 38 isolates (80%) (31 *bla*_IMP-1_, 5 *bla*_IMP-10_, and 2 *bla*_IMP-6_), followed by 3 *bla*_IMP-7_, 2 *bla*_IMP-11_ group (1 *bla*_IMP-11_ and 1 *bla*_IMP-41_), and 1 *bla*_VIM-1_, as anticipated (Table 2) [15-19]. While *bla*_IMP-1_ and *bla*_IMP-10_ were found both in 2004 and 2006, *bla*_IMP-7_, *bla*_IMP-11_, and *bla*_IMP-41_ emerged in 2006.

All MBL-producing isolates possessed *intI1*, and all MBL genes were located in the GC arrays of class 1 integron (Table 2); 16 distinct MBL-harboring integrons were identified. Complete GC arrays of all integrons except one were successfully sequenced, as many as 13 of which were novel to INTEGALL database. Fifteen isolates harbored In113-like integrons, which consisted of 3 GCs, namely *bla*_IMP-1_ group cassette (*bla*_IMP-1_ in In113, *bla*_IMP-6_ in In915, and *bla*_IMP-10_ in In829) followed by *aacA28* and *aacA1a* GCs. Similarly, thirteen isolates harbored In994-like integrons which contains a sole *bla*_IMP-1_ group cassette (*bla*_IMP-1_ in In994 and *bla*_IMP-10_ in In995). The family of *bla*_MBL_ genes and their position in integron GC arrays did not show significant differences in resistance profile (Table 2).

### Genotypes and their relationship with integrons among MBL-producing *P. aeruginosa*

Among MBL-producing *P. aeruginosa* isolates, nine different STs were identified by MLST: the most prevalent was ST235 (17 isolates, 39%) followed by ST357 (14 isolates, 32%) and ST244 (4 isolates, 9%) (Table 2). While these major 3 STs were found both in 2004 and 2006, the other 6 STs were found either in 2004 or in 2006.

Most integrons distributed in the specific genotypes of the isolates. In113-like integrons were found in ST235/pulsotype B isolates with significantly high frequency (13/13 vs 2/31; *P* < 0.0001), while partial *bla*_IMP-7_-harboring integron was exclusively found in ST235/pulsotype C (3/3 vs 0/41; *P* < 0.0001). While distributed across 5 geographic regions in total, ST235 was significantly associated with Kanto region (10/12 vs 7/32; *P* = 0.0003). ST235 was also associated with resistance to piperacillin (13/17 vs 7/27; *P* = 0.0017). Although not significant, a trend of increased ST235 in 2006 was observed (5/23 in 2004 vs 12/21 in 2006; *P* = 0.0291).

MBL-producing ST235 isolates seemed the dominant MDRP lineage in Japan as also reported worldwide [6,8-11,13]. As ST235 isolates are described as harboring a variety of resistance determinants including integrons, its diverse geographic distribution across Japan reflects part of the worldwide dissemination. Especially, isolates of ST235/pulsotype B with In113-like integron, recovered from 3 regions in the present study, were well characterized as a representative of the MDRP in Japan [15,16,19].

In our analysis, ST357 was significantly associated with In994-like integrons (11/14 vs. 3/30, *P* < 0.0001). ST357 is another widely disseminated lineage also found in the present study [4,7,16]. Concurrently, ST357 was found in Chugoku region with significantly higher frequency (11/14 vs 3/30, *P* < 0.0001), which might possibly reflect local transmission. However, ST357 harbored 5 different integrons in total, including 2 with *bla*_IMP-11_ group GCs emerged in 2006 (In826 and In991).

The significant association of specific genotypes and integrons implies that, in a relatively short period of time, dissemination and transmission of the preexisting resistant lineage, rather than horizontal gene transfer or recombination *in situ*, might largely explain their endemicity. Moreover, the genotypes of MBL-producers were unexpectedly similar to those in the central Europe with different resistant determinants, suggesting their possible advantage beyond other genotypes in the clinical settings [4]. Although the isolate number was small, the present study elucidated the emergence of ST357 isolates with novel integrons in distant geographic regions in 2006, as well as an emergence of the ST235/pulsotype C with partial integron harboring *bla*_IMP-7_ in Kanto region, which might suggest the increased complexity of endemic lineages. In addition, isolates with ST175 was recovered in Kyushu with significantly higher frequency (3/3 vs 5/41, *P* = 0.0042).

One of the limitations of the present study is that this is retrospective in nature and therefore the results are not expected to be directly applied to the current clinical situation. There are also several reports in Japan addressing a variety of integron structures of *P. aeruginosa* [15-20], and they seemed to contain identical or similar integrons to ones identified in the current study. Nevertheless, the findings here are important, since most of the integron structures were novel to INTEGRALL database [12]; this could be explained by the lack of comprehensive platform for integron structure and nomenclature before the development of INTEGRALL database until recently. Alternatively, this might reflect true heterogeneity and diversity of the integrons among clinical isolates, especially among non-predominant lineages. Moreover, MLST comparison of cross-sectional nationwide survey is much informative for understanding of the dynamics of drug-resistant organisms. In addition, this is the first report of the presence of novel *fosI* gene cassette other than *Mycobacterium* spp. [26], and further analysis regarding the actual transfer route beyond the boundary of the species is warranted. As genotyping including MLST and INTEGRALL has been increasingly applied for tracking the development of the drug resistance rationally, the data in the present study would provide a robust baseline to estimate the current trend and future diversification of the drug-resistant lineages [20]. Another shortcoming of this study is that horizontal gene transfer of *bla*_MLB_ genes, possibly located on plasmid or chromosome, was not experimentally addressed.

## Conclusions

The current study offers an important “snapshot” of the molecular characteristics and dynamics of MBL-producing *P. aeruginosa* lineages including ST235 and ST357 across Japan and provide critical information for the design and implementation of surveillance programs targeted to the discovery of multidrug resistant *P. aeruginosa*.

## Methods

### Bacterial isolates

A total of 1,988 *P. aeruginosa* isolates (996 isolates in 2004 and 992 in 2006), were retrospectively collected as a part of nationwide surveillance programs in Japan (Japan Antimicrobial Resistance Surveillance Participants Group) [27,28]. Briefly, up to 10 non-duplicated patient isolates of 10 organism groups including *P. aeruginosa* were collected for three months in 2004 and in 2006 from a total of 100 institutions in 8 geographic regions. MBL-producers were phenotypically identified by the double-disk synergy test using ceftazidime (30 μg) and sodium mercaptoacetate (30 μg) disks (Eiken Chemical, Inc., Tokyo, Japan) following the manufacturer’s instructions, and were subjected to further molecular epidemiological investigation [29]. Ethical approval has been obtained from Toho University School of Medicine Ethics Committee (2511925030).

### Antimicrobial susceptibility testing

Minimum inhibitory concentrations (MICs) were measured by the broth microdilution method of the Clinical and Laboratory Standards Institute (CLSI) [30]. The antimicrobial agents tested were as follows: piperacillin sodium salt, ceftazidime hydrate, aztreonam, imipenem monohydrate, amikacin sulfate salt, ciprofloxacin hydrochloride monohydrate (Sigma-Aldrich Japan KK., Tokyo, Japan), cefepime hydrochloride (Bristol-Myers Squibb. Tokyo, Japan), and arbekacin sulfate salt (Meiji Seika Pharma Co., Ltd. Tokyo, Japan). The antimicrobial concentration ranges were 0.06 - 128 μg/ml except for ciprofloxacin (0.25 - 512 μg/ml). MICs were interpreted as susceptible (S), intermediate (I), or resistant (R) according to CLSI interpretive criteria where available [31].

MDRP was defined as strains with non-susceptibility to at least 1 agent in ≥3 antimicrobial categories according to the international expert proposal [32]; amikacin as aminoglycosides, imipenem as antipseudomonal carbapenems, ceftazidime or cefepime as antipseudomonal cephalosporins, aztreonam as monobactams.

### Analysis of MBL genes and integron by PCR amplification and nucleotide sequencing

Genomic DNA was prepared by the Wizard genomic DNA preparation kit (Promega, Madison, WI, USA) according to the manufacturer’s recommendations. PCR was performed with Takara ExTaq (Takara Bio, Shiga, Japan). Nucleotide sequencing was performed by a commercial DNA sequencing service (Greiner Bio-one, Tokyo, Japan).

*bla*_MBL_ genes were detected and identified by PCR and nucleotide sequencing using primers for *bla*_IMP_ and *bla*_VIM_ [33]. *bla*_IMP-1_, *bla*_IMP-10_, and *bla*_IMP-6_ are referred as *bla*_IMP-1_ group because the deduced amino acid sequences of *bla*_IMP-1_ differ by single amino acid from those of *bla*_IMP-10_ and *bla*_IMP-6_. Similarly, *bla*_IMP-11_ and *bla*_IMP-41_ are as *bla*_IMP-11_ group because the deduced amino acid sequences differ by single amino acid. Integrase genes specific for class 1, 2, or 3 integrons were detected by PCR using primers by Shibata *et al.* [33]. Integron GC arrays were determined by PCR and nucleotide sequencing by primer walking [34]. According to INTEGRALL nomenclature, novel completely sequenced GCs, namely from a part of 5′-conserved segment (5′CS) to a part of 3′-conserved segment (3’CS), were submitted to INTEGRALL for the attribution of new integron numbers [12].

### Genotyping

MLST was performed as described by Curran *et al.* [3]. Pulsed-field gel electrophoresis (PFGE) of *Spe*I-digested DNA fragments from each strain was performed on a CHEF-MAPPER II apparatus (Bio Rad Laboratories, Rockland, Maine, USA) at 6 V/cm, for 19.7 h with pulse times running from 5.3 to 34.9 s in 0.5 × TBE buffer at 14°C. PFGE patterns in the dendrogram were analyzed with Molecular Analyst Software Fingerprinting II (Bio-Rad Laboratories). Only restriction fragments larger than 50 kb were used for analysis. PFGE genotypes were distinguished by the unweighted pair-group method using average linkages (UPGMA) and Dice coefficient, and isolates with ≥80% similarity were assigned into the same pulsotype.

### Statistical analyses

Statistical calculations were made using Graph-Pad Prism 5.01 (GraphPad Software Inc.). *P* values were calculated from two-tailed Fisher’s exact test for categorical variables, and *P* value <0.01 was considered as a statistically significant difference.

### Nucleotide sequences accession numbers for novel integrons

Genbank/EMBL/DDBJ accession numbers for novel nucleotide sequences in the present study are as follows: AB901034-AB901041 for In826-In833, and AB901042-AB901046 for In991-995, and AB901047 for partial integron of isolate TUM3995.

## References

[1] Queenan AM, Bush K (2007). Carbapenemases: the versatile β-lactamases. Clin Microbiol Rev.

[2] Partridge SR, Tsafnat G, Coiera E, Iredell JR (2009). Gene cassettes and cassette arrays in mobile resistance integrons. FEMS Microbiol Rev.

[3] Curran B, Jonas D, Grundmann H, Pitt T, Dowson CG (2004). Development of a multilocus sequence typing scheme for the opportunistic pathogen *Pseudomonas aeruginosa*. J Clin Microbiol.

[4] Nemec A, Krizova L, Maixnerova M, Musilek M (2010). Multidrug-resistant epidemic clones among bloodstream isolates of *Pseudomonas aeruginosa* in the Czech Republic. Res Microbiol.

[5] Garcia-Castillo M, Del Campo R, Morosini MI, Riera E, Cabot G, Willems R (2011). Wide dispersion of ST175 clone despite high genetic diversity of carbapenem-nonsusceptible *Pseudomonas aeruginosa* clinical strains in 16 Spanish hospitals. J Clin Microbiol.

[6] Maatallah M, Cheriaa J, Backhrouf A, Iversen A, Grundmann H, Do T (2011). Population structure of *Pseudomonas aeruginosa* from five Mediterranean countries: evidence for frequent recombination and epidemic occurrence of CC235. PLoS One.

[7] Hrabak J, Cervena D, Izdebski R, Duljasz W, Gniadkowski M, Fridrichova M (2011). Regional spread of *Pseudomonas aeruginosa* ST357 producing IMP-7 metallo-β-lactamase in Central Europe. J Clin Microbiol.

[8] Giske CG, Libisch B, Colinon C, Scoulica E, Pagani L, Fuzi M (2006). Establishing clonal relationships between VIM-1-like metallo-β-lactamase-producing *Pseudomonas aeruginosa* strains from four European countries by multilocus sequence typing. J Clin Microbiol.

[9] Empel J, Filczak K, Mrowka A, Hryniewicz W, Livermore DM, Gniadkowski M (2007). Outbreak of *Pseudomonas aeruginosa* infections with PER-1 extended-spectrum β-lactamase in Warsaw, Poland: further evidence for an international clonal complex. J Clin Microbiol.

[10] Yoo JS, Yang JW, Kim HM, Byeon J, Kim HS, Yoo JI (2012). Dissemination of genetically related IMP-6-producing multidrug-resistant *Pseudomonas aeruginosa* ST235 in South Korea. Int J Antimicrob Agents.

[11] Pournaras S, Kock R, Mossialos D, Mellmann A, Sakellaris V, Stathopoulos C (2013). Detection of a phylogenetically distinct IMP-type metallo-β-lactamase, IMP-35, in a CC235 *Pseudomonas aeruginosa* from the Dutch-German border region (Euregio). J Antimicrob Chemother.

[12] Moura A, Soares M, Pereira C, Leitao N, Henriques I, Correia A (2009). INTEGRALL: a database and search engine for integrons, integrases and gene cassettes. Bioinformatics.

[13] Edelstein MV, Skleenova EN, Shevchenko OV, D’Souza JW, Tapalski DV, Azizov IS (2013). Spread of extensively resistant VIM-2-positive ST235 *Pseudomonas aeruginosa* in Belarus, Kazakhstan, and Russia: a longitudinal epidemiological and clinical study. Lancet Infect Dis.

[14] Kataoka K, Ida T, Ishii Y, Tateda K, Oguri T, Yoshida A (2013). Analysis of the influence of drug resistance factors on the efficacy of combinations of antibiotics for multidrug-resistant *Pseudomonas aeruginosa* isolated from hospitals located in the suburbs of Kanto area, Japan. J Glob Antimicrob Resist.

[15] Kitao T, Tada T, Tanaka M, Narahara K, Shimojima M, Shimada K (2012). Emergence of a novel multidrug-resistant *Pseudomonas aeruginosa* strain producing IMP-type metallo-β-lactamases and AAC(6′)-Iae in Japan. Int J Antimicrob Agents.

[16] Kouda S, Ohara M, Onodera M, Fujiue Y, Sasaki M, Kohara T (2009). Increased prevalence and clonal dissemination of multidrug-resistant *Pseudomonas aeruginosa* with the *bla*_IMP-1_ gene cassette in Hiroshima. J Antimicrob Chemother.

[17] Zhao WH, Chen G, Ito R, Hu ZQ (2009). Relevance of resistance levels to carbapenems and integron-borne *bla*_IMP-1_, *bla*_IMP-7_, *bla*_IMP-10_ and *bla*_VIM-2_ in clinical isolates of *Pseudomonas aeruginosa*. J Med Microbiol.

[18] Kimura S, Alba J, Shiroto K, Sano R, Niki Y, Maesaki S (2005). Clonal diversity of metallo-β-lactamase-possessing *Pseudomonas aeruginosa* in geographically diverse regions of Japan. J Clin Microbiol.

[19] Sekiguchi J, Asagi T, Miyoshi-Akiyama T, Kasai A, Mizuguchi Y, Araake M (2007). Outbreaks of multidrug-resistant *Pseudomonas aeruginosa* in community hospitals in Japan. J Clin Microbiol.

[20] Notake S, Matsuda M, Tamai K, Yanagisawa H, Hiramatsu K, Kikuchi K (2013). Detection of IMP metallo-β-lactamase in carbapenem-nonsusceptible Enterobacteriaceae and non-glucose-fermenting Gram-negative rods by immunochromatography assay. J Clin Microbiol.

[21] Niki Y, Hanaki H, Matsumoto T, Yagisawa M, Kohno S, Aoki N (2011). Nationwide surveillance of bacterial respiratory pathogens conducted by the Japanese Society of Chemotherapy in 2008: general view of the pathogens’ antibacterial susceptibility. J Infect Chemother.

[22] Watanabe A, Yanagihara K, Matsumoto T, Kohno S, Aoki N, Oguri T (2012). Nationwide surveillance of bacterial respiratory pathogens conducted by the Surveillance Committee of Japanese Society of Chemotherapy, Japanese Association for Infectious Diseases, and Japanese Society for Clinical Microbiology in 2009: general view of the pathogens’ antibacterial susceptibility. J Infect Chemother.

[23] Tsuji A, Kobayashi I, Oguri T, Inoue M, Yabuuchi E, Goto S (2005). An epidemiological study of the susceptibility and frequency of multiple-drug-resistant strains of *Pseudomonas aeruginosa* isolated at medical institutes nationwide in Japan. J Infect Chemother.

[24] Yano H, Kuga A, Okamoto R, Kitasato H, Kobayashi T, Inoue M (2001). Plasmid-encoded metallo-β-lactamase (IMP-6) conferring resistance to carbapenems, especially meropenem. Antimicrob Agents Chemother.

[25] Araoka H, Baba M, Tateda K, Ishii Y, Oguri T, Okuzumi K (2012). In vitro combination effects of aztreonam and aminoglycoside against multidrug-resistant *Pseudomonas aeruginosa* in Japan. Jpn J Infect Dis.

[26] Leao SC, Matsumoto CK, Carneiro A, Ramos RT, Nogueira CL, Lima JD (2013). The detection and sequencing of a broad-host-range conjugative IncP-1β plasmid in an epidemic strain of *Mycobacterium abscessus* subsp. *bolletii*. PLoS One.

[27] Ishii Y, Alba J, Kimura S, Yamaguchi K (2006). Evaluation of antimicrobial activity of β-lactam antibiotics by Etest against clinical isolates from 100 medical centers in Japan (2004). Diagn Microbiol Infect Dis.

[28] Ishii Y, Tateda K, Yamaguchi K (2008). Evaluation of antimicrobial susceptibility for β-lactams using the Etest method against clinical isolates from 100 medical centers in Japan (2006). Diagn Microbiol Infect Dis.

[29] Arakawa Y, Shibata N, Shibayama K, Kurokawa H, Yagi T, Fujiwara H (2000). Convenient test for screening metallo-β-lactamase-producing gram-negative bacteria by using thiol compounds. J Clin Microbiol.

[30] Clinical and Laboratory Standards Institute (2012). Methods for dilution antimicrobial susceptibility tests for bacteria that grow aerobically -Ninth edition: approved standard M07-A9.

[31] Clinical and Laboratory Standards Institute (2012). Performance Standards for Antimicrobial Susceptibility Testing -Twenty-second Informational Supplement M100-S22.

[32] Magiorakos AP, Srinivasan A, Carey RB, Carmeli Y, Falagas ME, Giske CG (2012). Multidrug-resistant, extensively drug-resistant and pandrug-resistant bacteria: an international expert proposal for interim standard definitions for acquired resistance. Clin Microbiol Infect.

[33] Shibata N, Doi Y, Yamane K, Yagi T, Kurokawa H, Shibayama K (2003). PCR typing of genetic determinants for metallo-β-lactamases and integrases carried by gram-negative bacteria isolated in Japan, with focus on the class 3 integron. J Clin Microbiol.

[34] Martinez-Freijo P, Fluit AC, Schmitz FJ, Grek VS, Verhoef J, Jones ME (1998). Class I integrons in Gram-negative isolates from different European hospitals and association with decreased susceptibility to multiple antibiotic compounds. J Antimicrob Chemother.

